# Estimates and Predictions of Coal Workers’ Pneumoconiosis Cases among Redeployed Coal Workers of the Fuxin Mining Industry Group in China: A Historical Cohort Study

**DOI:** 10.1371/journal.pone.0148179

**Published:** 2016-02-04

**Authors:** Bing Han, Hongbo Liu, Guojiang Zhai, Qun Wang, Jie Liang, Mengcang Zhang, Kai Cui, Fuhai Shen, Hongbo Yi, Yuting Li, Yuhan Zhai, Yang Sheng, Jie Chen

**Affiliations:** 1 Department of Occupational and Environmental Health, School of Public Health, China Medical University, Shenyang, Liaoning, 110122, China; 2 Department of Health Statistics, School of Public Health, China Medical University, Shenyang, Liaoning, 110122, China; 3 Fuxin Mining Area Social Security Administration Center, Fuxin, Liaoning, 123000, China; 4 Institute for Occupational Disease Prevention and Treatment of the Fuxin Mining Industry Group, Fuxin, Liaoning, 123000, China; West Virginia University, UNITED STATES

## Abstract

This research was aimed at estimating possible Coal workers’ pneumoconiosis (CWP) cases as of 2012, and predicting future CWP cases among redeployed coal workers from the Fuxin Mining Industry Group. This study provided the scientific basis for regulations on CWP screening and diagnosis and labor insurance policies for redeployed coal workers of resource-exhausted mines. The study cohort included 19,116 coal workers. The cumulative incidence of CWP was calculated by the life-table method. Possible CWP cases by occupational category were estimated through the average annual incidence rate of CWP and males’ life expectancy. It was estimated that 141 redeployed coal workers might have suffered from CWP as of 2012, and 221 redeployed coal workers could suffer from CWP in the future. It is crucial to establish a set of feasible and affordable regulations on CWP screening and diagnosis as well as labor insurance policies for redeployed coal workers of resource-exhausted coal mines in China.

## Introduction

In China, there are 262 resource-based cities that prosper and grow due to natural resources [1–3]. Sixty-seven of them have been deemed resource-exhausted cities by the government [3]. Fuxin, located in northeast China, was first deemed a resource-exhausted city in 2001. Its leading industry had been coal [3, 4]. The majority of Fuxin’s mines had been gradually depleted since the 1980s, as major Fuxin coal mines had been exploited for over 100 years [4, 5]. The Fuxin Mining Industry Group is a state-owned enterprise and the major coal mine owner in Fuxin. Since 2001, several mines of the Fuxin Mining Industry Group have been exhausted, leading to Fuxin’s economic decline in the coal industry [4, 5]. The economic recession led to the layoff of more than one-third of all Fuxin workers in 2001. Of those unemployed, 45% were redeployed coal workers, which referred to coal workers who no longer worked for the Fuxin Mining Industry Group because of coal mines’ depletion [4, 5]. In 2001, the gross national product of Fuxin was only ¥3,700 per capita, which caused insufficient financial input into health and medical areas [4, 5]. Therefore, Fuxin was unable to provide redeployed coal workers from resource-exhausted coal mines with medical screening and labor insurance. The current administration authority of redeployed coal workers, Fuxin Mining Area Social Security Administration Center, does not provide screening and diagnosis of occupational diseases, which causes a regulatory gap for redeployed coal workers.

Coal workers’ pneumoconiosis (CWP) refers to the accumulation of coal dust in the lungs, causing local inflammation [6, 7]. CWP has been a major occupational disease internationally. Following implementation of the 1969 Federal Coal Mine Health and Safety Act [8], the prevalence of CWP among coal miners declined from approximately 30% in the early 1970s to 2% for the period of 1995–1999. But since 2000, the prevalence of CWP has increased [9, 10]. Previous research on 90,973 American coal workers found that the prevalence of CWP had increased to 3.1% from 1.7% since 1999 [11]. In the United Kingdom, the prevalence of CWP fell gradually from 12% in 1959–1963 to 0.2% in 1994–1997. In the years 1998–2000, the prevalence rose to 0.8% [12]. There were similar trends also in China. As of 2012, cases of pneumoconiosis rose to 727,148 in China. In 2012, 27,420 cases of occupational diseases were reported, of which 12,405 were CWP cases, accounting for 45.24% of all occupational disease cases. Currently, CWP is the most prevalent occupational disease in China [13]. Since CWP is a chronic and progressive occupational disease, continued treatments are a huge economic burden to CWP patients, their families, and the community [14, 15]. Previous estimations suggested pneumoconiosis caused economic costs of ¥184.5 billion, which accounted for 5.5‰ of China’s GDP for 2009 [16]. Therefore, pneumoconiosis has become a major obstacle for economic development and growth in China.

The current regulatory gap in occupational health management may lead to severe social conflicts. It also violates the 2011 Amendments to the Law of the People's Republic of China on Prevention and Control of Occupational Diseases [17]. Previous studies confirmed that even in coal workers no longer exposed to dust, residual dust in lungs still reacts with alveolar macrophages. Thus, redeployed coal workers may suffer from new cases of CWP [18–20]. Moreover, others could suffer from CWP in the future. Therefore, redeployed coal workers need the attention of authorities and the public.

This study aimed at estimating the possible CWP cases as of 2012, and predicting future CWP cases among redeployed coal workers from resource-exhausted mines of the Fuxin Mining Industry Group.

## Study Population and Methods

### Study population

The study cohort was conducted in 2012 in the Fuxin Mining Industry Group at a total of ten mines, of which five mines were resource-exhausted coal mines and five mines were working coal mines. The redeployed coal workers ceased exposure to dust after their redeployment. All related information on coal workers included personnel files, individual medical records, and occupational records between 1 January 1965 and 31 December 2012. We collected historical data on work history and diagnosed pneumoconiosis as of 2012. The study was approved by the Medical Ethics Committee of China Medical University (permit number CMU6206-3008). The need for written consent was not deemed necessary and was waived by the Medical Ethics Committee. The study was carried out in accordance with approved guidelines.

The investigated coal workers were included in this study as long as they had dust exposure since 1965. The selection criteria of the study subjects were as follows: 1) the duration of dust exposure should be at least one year; 2) employment records of subjects should be complete and able to reflect job shifts.

The database included demographic details, work history record with the date of dust exposure, and individual medical and pneumoconiosis diagnosis records. For coal workers, observed years started on the first day of dust exposure and ended on the date when the worker was lost to follow-up, or the study ended (31 December 2012). For the CWP patients, observed years started on the first day of dust exposure and ended on the date when they were diagnosed with CWP. The date of diagnosis of CWP was included in the database. The information on redeployed coal workers were obtained from the Fuxin Mining Area Social Security Administration Center. The information on current coal workers and CWP patients were obtained from the Institute for Occupational Disease Prevention and Treatment of the Fuxin Mining Industry Group.

### Diagnosis of pneumoconiosis

The diagnosis of CWP was based on the “Diagnostic criteria of pneumoconiosis” and corresponding standard films of pneumoconiosis in China [21]. The Chinese “Diagnostic criteria of pneumoconiosis” was formulated based on the “Guidelines for the use of the International Labor Organization international classification of radiographs of pneumoconiosis” [22]. The diagnosis of pneumoconiosis was based on Chinese diagnostic criteria of pneumoconiosis and corresponding standard films of pneumoconiosis, and was made by five qualified experts, who were all members of the Pneumoconiosis Diagnosis Committee, with the records of historical exposure to dust and chest X-ray [21, 22].

### Occupational category

Occupational categories were defined as tunneling, mining, combining, or helping coal workers. The duration of dust exposure for each coal worker was the sum of years of each dust exposure job. The duration of each dust exposure job was measured from the start date to the end date of the job. Coal workers who worked several jobs were classified as tunneling if their tunneling time was more than half of the whole duration of dust exposure. They would be classified as mining workers if they were involved in tunneling for less than two years and their mining duration was more than half of the whole duration of dust exposure. Combining workers were those whose tunneling time was more than two years but not more than half of the whole duration of dust exposure. Helping workers were those who did not fit in any of the tunneling, mining, or combining categories [22–24].

### Calculation of the cumulative incidence rate

Four subcohorts were established according to occupational category: tunneling, mining, combining, and helping. Applying the life-table method and stratified analysis, the CWP cumulative incidence of each subcohort was calculated in the corresponding observed years. The average annual incidence rate of CWP was defined as the rate of cumulative incidence and observed years (Eq 1) [22, 23]. We calculated the cumulative incidence rates, using the 1965- tunneling subcohort as an example in S1 Table. Cumulative incidence rates of the other cohorts were calculated in the same manner.

$${\bar{P}}_{ac}=P_{ac}/T_{ac}$$ (1)

In the formula, ${\bar{P}}_{ac}$ is the average annual incidence rate of CWP (‰); *P*_*ac*_ is the CWP cumulative incidence of each subcohort (%); *T*_*ac*_ is the observed years; *a* is the year of first dust exposure (1965- and 1975-); *c* is the occupational category (tunneling, mining, combining, and helping).

### Estimates of possible CWP cases among redeployed coal workers as of 2012

#### Estimated possible CWP cases by age group

Redeployed coal workers were divided into groups of five-year by age. Taking the average annual incidence rate of CWP in each subcohort as annual incidence, and life expectation of males in Fuxin as the redeployed coal workers’ life expectation, numbers of possible CWP cases as of 2012 were estimated by year of first dust exposure and occupational categories (Eqs 2, 3 and 4). Previous studies suggest that the above method is precise and suitable for the calculations [22, 23].

$$N_{acr}=x_{acr}.E_{r}.{\bar{P}}_{ac}$$ (2)

$$N_{c}=\sum_{a=1}^{g}\sum_{r=1}^{f}N_{acr}$$ (3)

$$N_{r}=\sum_{a=1}^{g}\sum_{c=1}^{m}N_{acr}$$ (4)

In the formula, *N*_*acr*_ is the numbers of possible CWP cases of every age group; *x*_*acr*_ is the numbers of redeployed coal workers of every age group; *E*_*r*_ is the life expectation of Fuxin males of every age group; *r* is the age group; *N*_*c*_ is the numbers of possible CWP cases of different occupational category; *N*_*r*_ is the total numbers of possible CWP cases of every age group.

#### Estimated possible CWP cases by time period

Redeployed coal workers were divided into groups of five-year by time period. Taking the average annual incidence rate of CWP of each subcohort as annual incidence, and life expectation of males in Fuxin as the redeployed coal workers’ life expectation, numbers of possible CWP cases were estimated by year of first dust exposure and occupational category (Eqs 5 and 6).

$$N_{ack}=\sum_{r=1}^{f}x_{acr}.t_{k}.{\bar{P}}_{ac}$$ (5)

$$If2k-E_{\text{i}}>2,t_{k}=2;if0<2k-E_{i}<2,t_{k}=E_{i}-2k;if2k-E_{i}<0,t_{k}=0$$

$$N_{k}=\sum_{a=1}^{g}\sum_{c=1}^{m}N_{ack}$$ (6)

In the formula, *N*_*ack*_ is the numbers of possible CWP cases of every time period; *x*_*acr*_ is the numbers of redeployed coal workers of every age group; *t*_*k*_ is the time period, *k* = 1,2,…,*q*; *E*_i_ is the life expectation of males of every age group in Fuxin; *N*_*k*_ is the total numbers of possible CWP cases among redeployed coal workers.

### Predictions of future CWP cases among redeployed coal workers

#### Predicted future CWP cases by age group

Numbers of future CWP cases among redeployed coal workers by the year of first dust exposure and occupational category since 1 January 2013 were predicted by five-year age group, using life expectation and average annual incidence by subcohorts of redeployed coal workers (Eqs 2, 3 and 4).

#### Predicted future CWP cases by time period

Numbers of future CWP cases among redeployed coal workers by the year of first dust exposure and occupational category since 1 January 2013 were predicted with five-year time period, using life expectation and average annual incidence by subcohorts of redeployed coal workers (Eqs 5 and 6).

## Results

### Baseline characteristics

The study investigated 19,116 male coal workers who had either worked or were working in coal mines with over one year dust exposure. Of all subjects, 9,449 were redeployed coal workers, 9,256 were working coal workers, and 411 were CWP patients. The median dust exposure duration of patients with CWP was 22.67 years, and the median of observed years was 24.00 years as of 2012; the median dust exposure duration of coal workers with dust exposure was 20.76 years, and the median of observed years was 24.00 years as of 2012 (Table 1).

**Table 1:** Baseline characteristics of coal workers studied.

Variable	Patients with CWP	Coal workers with dust exposure
	(n = 411)	(n = 18,705)
Alive		
Yes	384	18,535
No	27	170
Redeployed		
Yes	411	9,449
No	0	9,256
Occupational category		
Tunneling	295	5,734
Mining	95	4,061
Combining	12	1,325
Helping	8	7,585
Age	46.02(29.61–59.44) ^a^ ^c^	52.57(20.42–88.71) ^b^ ^c^
Year of first dust exposure		
1965-	239	4,467
1975-	172	14,238
Duration of dust exposure	22.67 (3.34–37.43) ^c^	20.76 (1.08–44.53) ^c^
Observed years	24.00 (6.29–38.56) ^c^	24.00 (1.00–45.40) ^c^

^a^ the median diagnostic age of CWP patients.

^b^ the median age of all coal workers with dust exposure as of 2012.

^c^ the table cell showed the estimated median (Min-Max) combination.

### Estimated possible CWP cases among redeployed coal workers as of 2012

#### Estimated possible CWP cases by age group

This study investigated 9,449 redeployed coal workers with dust exposure, of which 9,279 were alive when the study finished. The life-table method with stratified adjustment was used to estimate possible CWP cases among surviving redeployed coal workers. It was assumed that the life expectancy of workers in different subcohorts equaled that of Fuxin’s male residents. Based on average annual incidence (S2 Table), age distribution according to year of first dust exposure and occupational category (S3 Table), and the life expectancy of coal workers, possible CWP cases according to year of first dust exposure and occupational category as of 2012 were respectively calculated (Table 2). The result suggested that 141 redeployed coal workers might have suffered from CWP. Of all possible CWP cases, 97 (68.79%) tunneling, 35 (24.82%) mining, 4 (2.84%) combining, and 5 (3.55%) helping redeployed coal workers were estimated to have suffered from CWP.

**Table 2:** Possible CWP cases among redeployed coal workers by age group as of 2012.

Years of first dust exposure	Occupational category	Age group										
		25-	30-	35-	40-	45-	50-	55-	60-	65-	70-	Total
1965-	Tunneling	-	-	-	0.51	19.59	27.04	20.16	2.22	0.76	0.50	70.78
	Mining	-	-	-	0.09	6.76	10.43	8.76	1.13	0.55	0.18	27.89
	Combining	-	-	-	-	0.53	1.39	0.93	0.15	0.11	0.01	3.12
	Helping	-	-	-	0.02	1.90	1.39	0.64	0.22	0.22	0.07	4.46
1975-	Tunneling	0.10	0.88	4.97	9.27	9.20	1.52	0.49	0.14	0.09	-	26.67
	Mining	0.11	0.49	1.50	1.82	2.16	0.44	0.18	0.04	0.02	-	6.78
	Combining	-	0.01	0.13	0.12	0.13	0.02	-	-	-	-	0.41
	Helping	-	0.02	0.19	0.19	0.18	0.04	0.01	0.01	0.01	-	0.63
Total		0.21	1.40	6.79	12.02	40.45	42.27	31.17	3.91	1.76	0.76	140.74

#### Estimated possible CWP cases by time period

**Fig 1:**
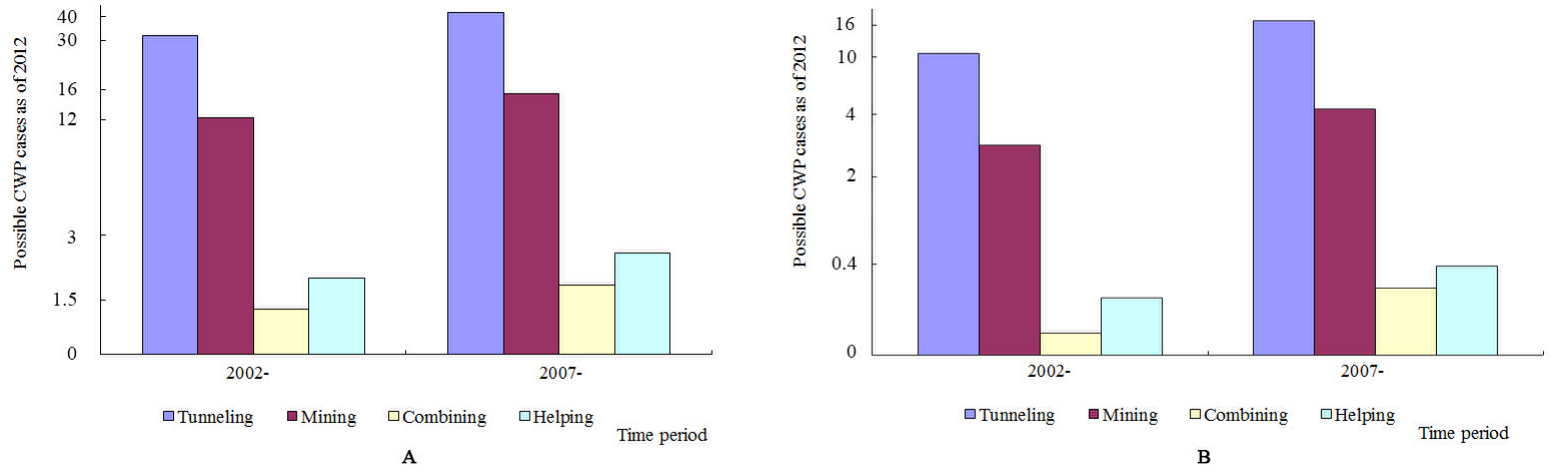
Possible CWP cases among redeployed coal workers by time period as of 2012. A: Possible CWP cases among redeployed coal workers of 1965-; B: Possible CWP cases among redeployed coal workers of 1975-.

As Fig 1 shows, as of 2012, 59 (41.84%) redeployed coal workers suffered from CWP during 2002–2006, and 82 (58.16%) suffered from CWP during 2007–2012.

Subcohorts of tunneling, mining, combining, and helping coal workers who suffered from CWP were respectively 57, 20, 2, and 3 during 2007–2012, which were all higher than those with CWP during 2002–2006 (41, 16, 1, and 2, respectively).

### Predicted future CWP cases among redeployed coal workers

#### Predicted future CWP cases by age group

As shown in Table 3, 221 redeployed coal workers would suffer from CWP in the future. Of all future CWP cases, there would be 155 (70.14%) tunneling, 53 (23.98%) mining, 4 (1.81%) combining, and 8 (3.63%) helping redeployed coal workers.

**Table 3:** Possible future CWP cases among redeployed coal workers by age group.

Years of first dust exposure	Occupational category	Age groups									
		25-	30-	35-	40-	45-	50-	55-	60-	65-	Total
1965-	Tunneling	-	-	-	1.07	36.85	35.19	18.79	1.37	0.21	93.48
	Mining	-	-	-	0.20	13.26	13.54	8.16	0.70	0.15	36.01
	Combining	-	-	-	-	1.14	1.78	0.87	0.09	0.03	3.91
	Helping	-	-	-	0.04	3.49	1.83	0.59	0.14	0.06	6.15
1975-	Tunneling	0.71	3.29	14.18	22.71	18.16	2.04	0.46	0.11	-	61.66
	Mining	0.61	1.82	4.36	4.69	4.48	0.58	0.17	0.03	-	16.74
	Combining	0.01	0.02	0.38	0.33	0.29	0.03	-	-	-	1.06
	Helping	0.02	0.07	0.54	0.46	0.34	0.05	0.01	0.01	-	1.50
Total		1.35	5.20	19.46	29.50	78.01	55.04	29.05	2.45	0.45	220.51

#### Predicted future CWP cases by time period

**Fig 2:**
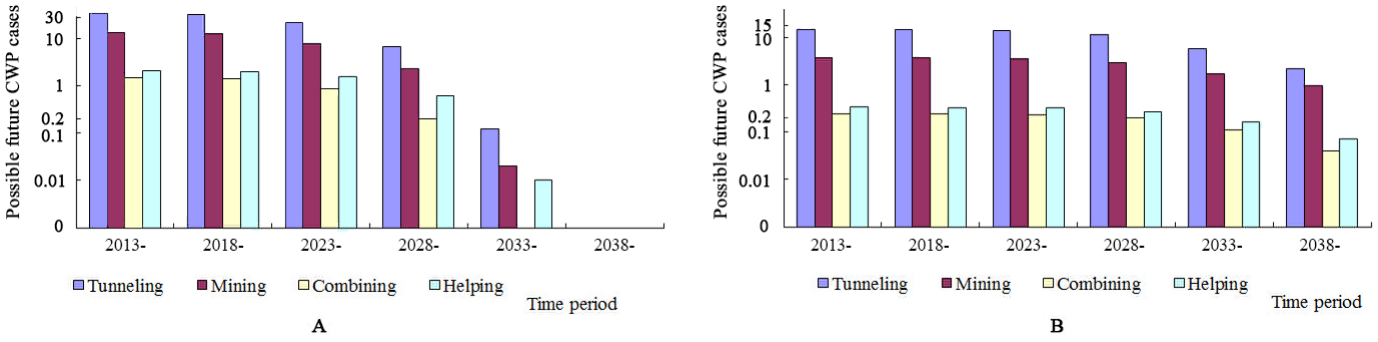
Possible future CWP cases among redeployed coal workers by time period. A Possible future CWP cases among redeployed coal workers of 1965-; B Possible future CWP cases among redeployed coal workers of 1975-.

As Fig 2 shows, the time period with the most future CWP cases in 30 years would be 2013–2028, and in that time period, 185 (83.71%) redeployed coal workers would suffer from CWP. After 2028, the redeployed coal workers investigated would gradually reach their life expectancy, and new cases would decrease to 36 (16.29%).

During 2013–2027, tunneling, mining, combining, and helping redeployed coal workers who would suffer from CWP would be respectively 129, 45, 4, and 7. After 2028, the numbers would be respectively 26, 8, 1, and 1.

## Discussions

Coal is the major strategic energy source of China, accounting for more than 60% of primary energy production and consumption [13, 25]. Coal industry provides over 6 million jobs in China, which indicates the largest group of CWP patients in the world [13, 25]. Previous research on CWP found that CWP cases would still occur after coal workers’ cessation of dust exposure [18–20, 26]. Currently, regulatory gaps in Fuxin cause the lack of CWP screening and diagnosis for redeployed coal workers from resource-exhausted mines of the Fuxin Mining Industry Group.

Based on the analysis of the redeployed coal workers in resource-exhausted mines, regardless of the age group or time period, tunneling coal workers were the most likely to suffer from CWP among all occupational categories of possible cases as of 2012 or possible future cases. The results suggested that even when coal workers ceased exposure to high dust concentrations, they were still likely to suffer from CWP in the future [18–20, 26]. Denser SiO_2_ concentrations and longer working time were also two major factors in CWP occurrence [27, 28]. Therefore, tunneling workers should be the major concern of CWP screening and diagnosis among the redeployed coal workers. At the same time, this study found that 185 redeployed coal workers could suffer from CWP in the following 15 years. Because these redeployed coal workers would gradually reach their life expectancy, possible CWP cases would decrease after 2028. Therefore, in the following 15 years, there would be a peak of new CWP cases among those coal workers, and screening and diagnosis of CWP should be done immediately. However, current occupational disease authorities did not cover redeployed coal workers, and a regulatory gap of CWP screening and diagnosis occurred.

A series of factors consisted of the regulatory gap for redeployed coal workers of resource-exhausted mines in Fuxin. Firstly, Fuxin’s economy highly relied on the coal industry, and great numbers of coal workers were redeployed when there was a downturn in the coal industry due to resource exhaustion and a decline in the economy [29, 30]. Secondly, the Fuxin Mining Industry Group encountered many difficulties including historical economic burden due to organizational structures, ineffectiveness in coal manufacturing, and downturn in coal price [4, 5]. Therefore, it is impossible to afford CWP screening and diagnosis for redeployed coal workers. Thirdly, the coal industry did not require much education. Generally, redeployed coal workers were less educated, and lacked knowledge of CWP prevention. Subsequent jobs of redeployed coal workers were usually not well-paid, so the workers would not visit clinics when they showed CWP symptoms such as chest pain and congestion, shortness of breath, cough, and so forth.

Above factors caused the regulatory gap, which needs to be covered by policy-making. Thus, we suggest that redeployed coal workers of resource-exhausted coal mines need immediate CWP screening and diagnosis, as well as corresponding social securities, in order to fulfill the aim of prevention and control of pneumoconiosis proposed by the National Occupational Disease Control Program of China (2009–2015) [31]. There are a large number of redeployed coal workers in 67 resource-exhausted cities in China. Therefore, in addition to pre-employment and employment prevention and control of CWP, CWP screening and diagnosis among redeployed coal workers of resource-exhausted coal mines should be highly valued by the nation, authorities, and the public. It is crucial to establish a set of feasible and affordable regulations on CWP screening and diagnosis and social securities for redeployed coal workers of resource-exhausted coal mines in China.

This study had several limitations. Firstly, this research aimed to provide scientific evidence for authorities through estimates of possible CWP cases as of 2012, and predictions of future CWP cases; therefore, the study did not analyze related economic costs and welfare losses due to CWP, which would be addressed in further studies. Secondly, the research mainly studied redeployed underground coal workers, but redeployed surface coal workers were not included. In China, the majority of coal mines are underground, so the study focused on redeployed underground coal workers. We will conduct further research on redeployed surface coal workers in the near future.

## Conclusion

This study estimated that 141 redeployed coal workers might have suffered from CWP as of 2012. It was predicted that 221 redeployed coal workers could suffer from CWP in the future. Current occupational disease authorities did not cover redeployed coal workers. Therefore, it is crucial to establish a set of feasible and affordable regulations on CWP screening and diagnosis as well as social securities for redeployed coal workers of resource-exhausted coal mines. The study fits in the framework of prevention and control of occupational diseases for 67 resource-exhausted cities, and serves as a reference for other resource-based cities in China.

## Supporting Information

S1 Table Cumulative incidence rate of CWP in the 1965- tunneling subcohort. (DOC)

S2 Table Cumulative incidents and average annual incidence of coal workers by year of first dust exposure and occupational category. (DOC)

S3 Table Age distribution of redeployed coal workers by year of first dust exposure and occupational category. (DOC)

S1 File Supplementary Raw anonymized dataset. (XLS)
