# Supplementary material for: Estimates and Predictions of Coal Workers’ Pneumoconiosis Cases among Redeployed Coal Workers of the Fuxin Mining Industry Group in China: A Historical Cohort Study
Source: PLoS One. 2016 Feb 4;11(2):e0148179. doi: 10.1371/journal.pone.0148179 (PMC4742233; doi:10.1371/journal.pone.0148179)
Supplement: S1 Table — (DOC) [file pone.0148179.s001.doc]

Table S1. Cumulative incidence rate of CWP in the 1965- tunneling subcohort

| Observed years | Adjusted observed number | Incidence number | Incidence rate | No incidence rate | Cumulative no incidence rate | Cumulative incidence rate |
| --- | --- | --- | --- | --- | --- | --- |
| 0- | 1537 | 0 | 0.0000 | 1.0000 | 1.0000 | 0.0000 |
| 2- | 1537 | 0 | 0.0000 | 1.0000 | 1.0000 | 0.0000 |
| 4- | 1537 | 0 | 0.0000 | 1.0000 | 1.0000 | 0.0000 |
| 6- | 1537 | 0 | 0.0000 | 1.0000 | 1.0000 | 0.0000 |
| 8- | 1536 | 0 | 0.0000 | 1.0000 | 1.0000 | 0.0000 |
| 10- | 1533.5 | 0 | 0.0000 | 1.0000 | 1.0000 | 0.0000 |
| 12- | 1531 | 0 | 0.0000 | 1.0000 | 1.0000 | 0.0000 |
| 14- | 1527 | 2 | 0.0011 | 0.9989 | 0.9989 | 0.0011 |
| 16- | 1518.5 | 10 | 0.0054 | 0.9946 | 0.9935 | 0.0065 |
| 18- | 1496 | 19 | 0.0102 | 0.9898 | 0.9834 | 0.0166 |
| 20- | 1425.5 | 22 | 0.0141 | 0.9859 | 0.9695 | 0.0305 |
| 22- | 1296.5 | 21 | 0.0164 | 0.9836 | 0.9537 | 0.0463 |
| 24- | 1086.5 | 26 | 0.0243 | 0.9757 | 0.9305 | 0.0695 |
| 26- | 821 | 25 | 0.0305 | 0.9695 | 0.9021 | 0.0979 |
| 28- | 573.5 | 12 | 0.0208 | 0.9792 | 0.8833 | 0.1167 |
| 30- | 375 | 16 | 0.0424 | 0.9576 | 0.8458 | 0.1542 |
| 32- | 227.5 | 6 | 0.0266 | 0.9734 | 0.8233 | 0.1767 |
| 34- | 149.5 | 2 | 0.0134 | 0.9866 | 0.8123 | 0.1877 |
| 36-38 | 118 | 2 | 0.0168 | 0.9832 | 0.7986 | 0.2014 |
