# Supplementary material for: Estimates and Predictions of Coal Workers’ Pneumoconiosis Cases among Redeployed Coal Workers of the Fuxin Mining Industry Group in China: A Historical Cohort Study
Source: PLoS One. 2016 Feb 4;11(2):e0148179. doi: 10.1371/journal.pone.0148179 (PMC4742233; doi:10.1371/journal.pone.0148179)
Supplement: S2 Table — (DOC) [file pone.0148179.s002.doc]

Table S2. Cumulative incidents and average annual incidence of coal workers according to year of first dust exposure and occupational category

| Years of first dust exposure | Occupational category | Number of coal workers | Incidents | Observed years | Cumulative incidence (%) | Average annual incidence (‰) |
| --- | --- | --- | --- | --- | --- | --- |
| 1965- | Tunneling | 1374 | 163 | 38 | 20.14 | 5.2991 |
|  | Mining | 979 | 60 | 38 | 11.13 | 2.9279 |
|  | Combining | 250 | 9 | 38 | 5.11 | 1.3447 |
|  | Helping | 1864 | 7 | 38 | 0.90 | 0.2378 |
| 1975- | Tunneling | 4360 | 132 | 36 | 7.66 | 2.1290 |
|  | Mining | 3082 | 35 | 36 | 3.51 | 0.9736 |
|  | Combining | 1075 | 3 | 36 | 0.51 | 0.1411 |
|  | Helping | 5721 | 2 | 36 | 0.09 | 0.0258 |
| Total | | 18705 | 411 | - | - | - |
