# Supplementary material for: Estimates and Predictions of Coal Workers’ Pneumoconiosis Cases among Redeployed Coal Workers of the Fuxin Mining Industry Group in China: A Historical Cohort Study
Source: PLoS One. 2016 Feb 4;11(2):e0148179. doi: 10.1371/journal.pone.0148179 (PMC4742233; doi:10.1371/journal.pone.0148179)
Supplement: S3 Table — (DOC) [file pone.0148179.s003.doc]

Table S3. Age distribution of redeployed coal workers according to year of first dust exposure and occupational category

| Years of first dust exposure | Occupational category | Age group | | | | | | | | | | |
| --- | --- | --- | --- | --- | --- | --- | --- | --- | --- | --- | --- | --- |
|  |  | 25- | 30- | 35- | 40- | 45- | 50- | 55- | 60- | 65- | 70- | Total |
| 1965- | Tunneling | - | - | - | 9 | 384 | 471 | 346 | 38 | 13 | 9 | 1270 |
|  | Mining | - | - | - | 3 | 250 | 328 | 272 | 35 | 17 | 6 | 911 |
|  | Combining | - | - | - | - | 47 | 94 | 63 | 10 | 7 | 1 | 222 |
|  | Helping | - | - | - | 8 | 811 | 547 | 244 | 84 | 86 | 28 | 1808 |
| 1975- | Tunneling | 9 | 48 | 244 | 474 | 471 | 68 | 21 | 6 | 4 | - | 1345 |
|  | Mining | 17 | 58 | 164 | 214 | 254 | 42 | 17 | 4 | 2 | - | 772 |
|  | Combining | 1 | 5 | 98 | 105 | 112 | 13 | 2 | 1 | 1 | - | 338 |
|  | Helping | 21 | 89 | 765 | 792 | 726 | 139 | 33 | 29 | 19 | - | 2613 |
| Total |  | 48 | 200 | 1271 | 1605 | 3055 | 1702 | 998 | 207 | 149 | 44 | 9279 |
